# Preexisting Mental Disorders Increase the Risk of COVID-19 Infection and Associated Mortality

**DOI:** 10.3389/fpubh.2021.684112

**Published:** 2021-08-09

**Authors:** Yongjun Wang, Yang Yang, Lina Ren, Yuan Shao, Weiqun Tao, Xi-jian Dai

**Affiliations:** ^1^Shenzhen Mental Health Centre, Shenzhen Kangning Hospital, Shenzhen, China; ^2^Department of Radiology, Suining Central Hospital, Suining, China

**Keywords:** COVID-19, mental disorders, SARS-CoV-2, dementia, Parkinson's disease, late-life

## Abstract

Coronavirus disease 2019 (COVID-19), a respiratory disease of unknown origin, has a high rate of morbidity and mortality. Individuals with mental disorders may have a higher risk of infection and worse clinical outcomes because of a variety of factors such as poorer general resilience and lower immune function. However, there have been no studies to date specifically investigating the risk of COVID-19 and associated mortality in these patients. This was addressed in the present study by analyzing the data of 473,958 subjects included in the UK Biobank, 14,877 of whom tested positive for COVID-19 infection. Logistic regression analysis was performed to evaluate the associations between mental disorders and risks of COVID-19 infection and associated mortality. The results showed that subjects who were diagnosed with a mental disorder had a significantly higher risk of developing COVID-19 and a worse outcome as evidenced by higher rates of COVID-19-related mortality, with the strongest effects observed for dementia. Among dementia subtypes, Alzheimer disease patients had the highest risks of COVID-19 infection (7.39-fold increase) and associated mortality (2.13-fold increase). Late-life anxiety only increased the risk of developing COVID-19 while late-life depression not only was associated with a higher risk of infection but also a worse outcome. These findings highlight the need to prioritize patients with mental disorders-especially those who experience these disorders later in life—when implementing preventive strategies such as vaccinations.

## Introduction

Coronavirus disease 2019 (COVID-19), a respiratory disease of unknown origin caused by severe acute respiratory syndrome coronavirus 2 (SARS-CoV-2), has a high rate of morbidity and mortality, affecting 163 million people and resulting in 3.3 million deaths in over 200 countries (https://www.worldometers.info/coronavirus/, accessed on 16th May 2021). Although neutralizing antibody rapidly develops after infection (1), a second episode of asymptomatic infection occurring 142 days after the first symptomatic episode in an apparently immunocompetent patient has been recently reported (2), suggesting that COVID-19 is a greater public health threat than initially assumed.

According to the World Health Organization, about 20–25% of the adult population is affected by mental disorders (https://www.who.int/). These individuals may have a higher risk of various diseases and have worse physical health and treatment outcomes (3–5) because of a variety of factors such as shorter life expectancy, poorer general resilience, lower immunity (6, 7), and higher susceptibility to infection (8). Recent studies have reported psychological risk factors for infections in general population and among frontline healthcare workers (9, 10). Although several risk factors for COVID-19 infection and associated mortality have been documented [e.g., older age and the presence of complication; (11–13)], the risk for COVID-19 infection and associated mortality left largely unknown.

Recent studies have shown that individuals with a diagnosis of a mental disorder had a higher risk for COVID-19 infection and associated mortality. Li et al. found that patients with a psychiatric diagnosis had a higher risk of mortality compared with those without a psychiatric diagnosis, but the risk of subtypes of psychiatric diagnosis was not studied (14). Nemani et al. found that individuals with a recent psychiatric diagnosis of a schizophrenia spectrum was significantly associated with a higher risk of COVID-19 associated mortality in a small sample-sized cohort study, but those individuals with diagnoses of mood disorders and anxiety disorders were not associated with mortality (15). In a larger sample-sized cohort study by Wang et al. they found that individuals with a recent psychiatric diagnosis of an attention-deficit/hyperactivity disorder, a bipolar disorder, a depression and a schizophrenia had a significantly higher risk for COVID-19 infection (16). However, no such large sample-sized cohort studies concerning on systematically investigated the association of mental disorders (e.g., subtypes of dementia, anxiety, and depression) with COVID-19 infection and associated mortality, especially in a study.

Identifying high-risk populations is important as they can be prioritized in intervention strategies and receive timely and appropriate medical care. In the present larger sample-sized cohort study, we systematically evaluated the impact of middle aged and late-life adults diagnosed with a mental disorder—including psychotic disorders, substance use disorder, bipolar disorder, dementia, anxiety, depression, and depression comorbid with anxiety—on the risk for COVID-19 infection and associated mortality after adjusting for several confounding factors.

## Materials and Methods

### Subjects

**Figure 1:**
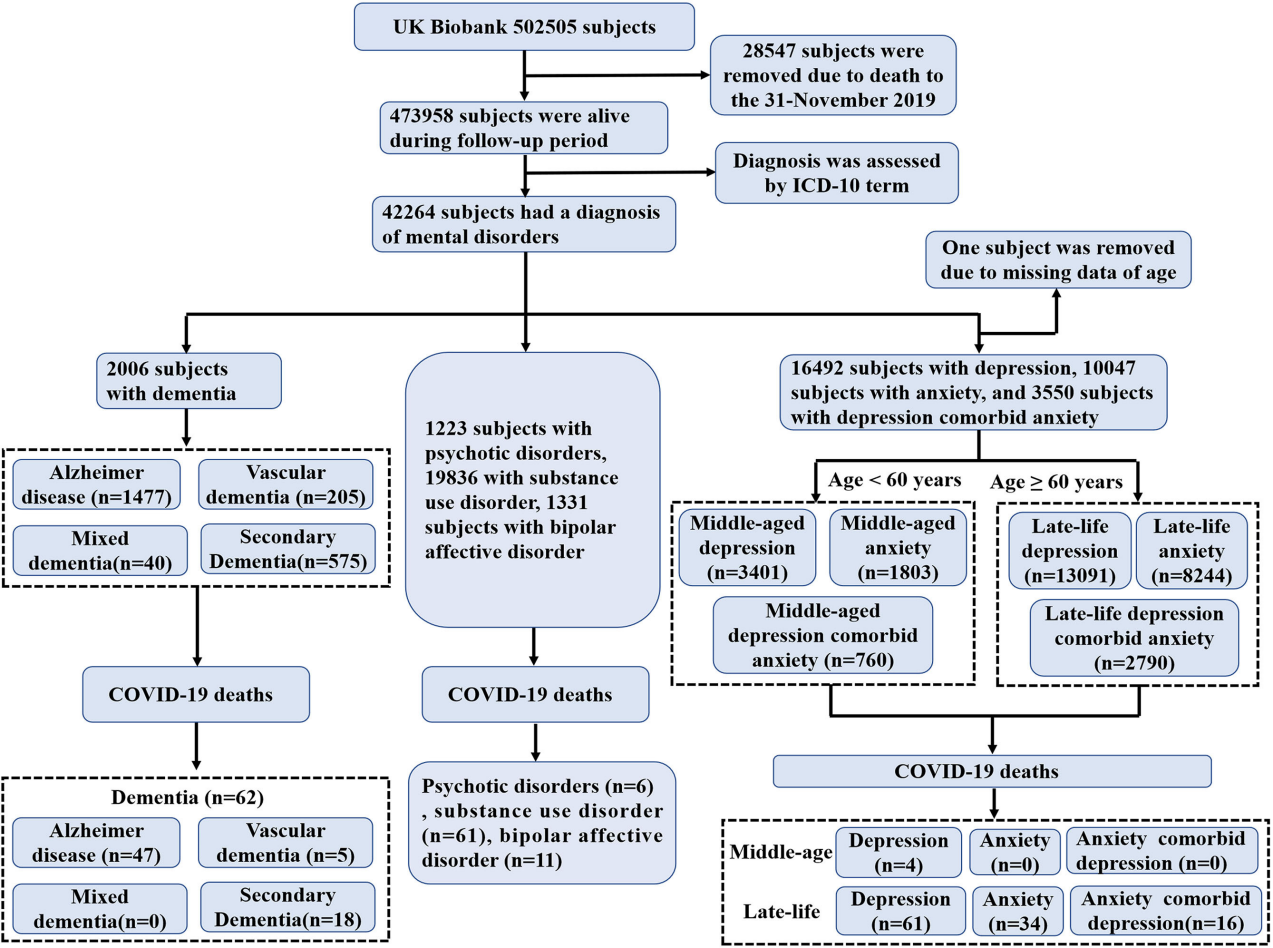
Overview of study sample derivation.

**Figure 2:**
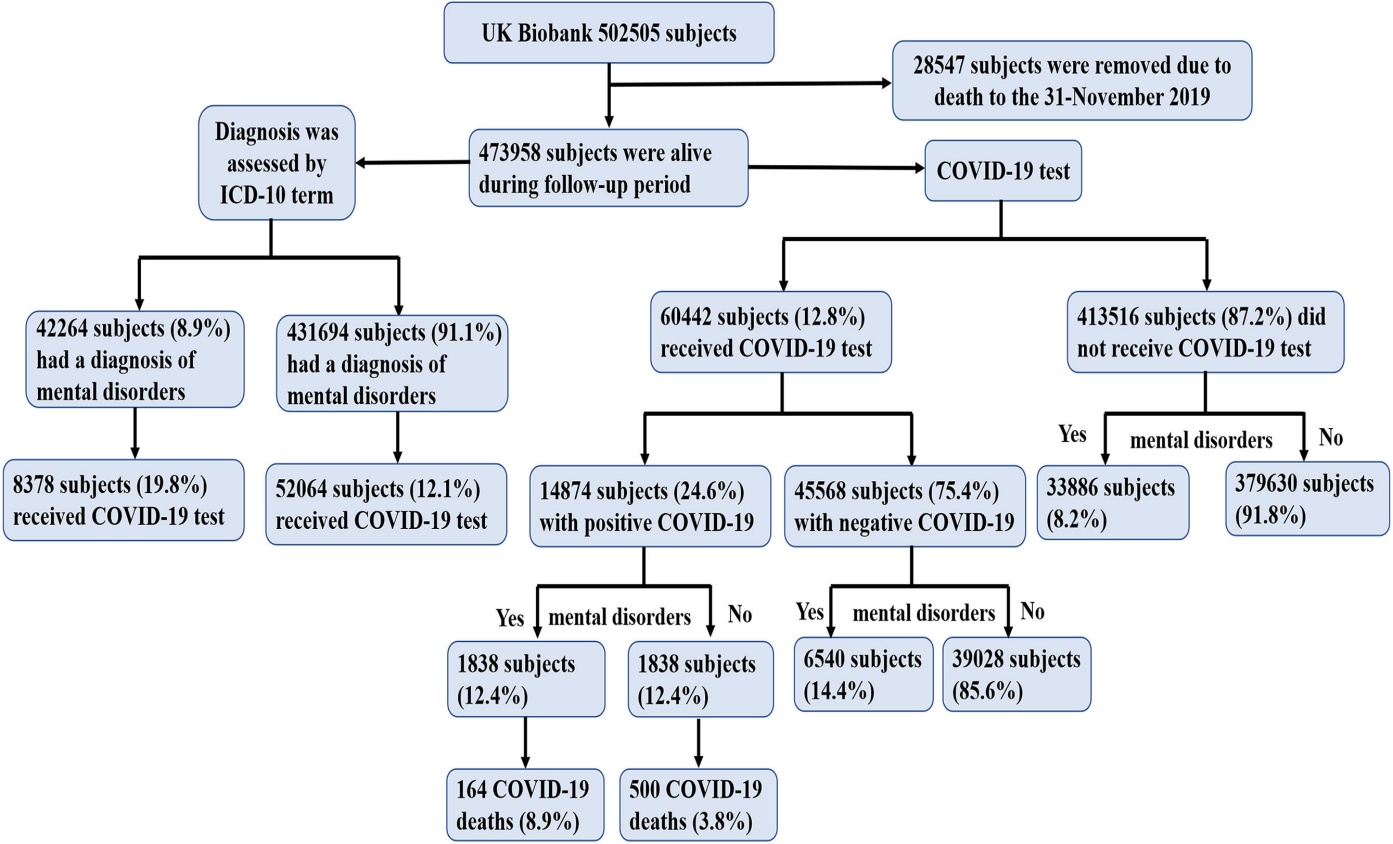
Participant flow diagram with COVID-19.

After removing the death data (*n* = 28,547) before 31-November 2019, the remaining data of 473,958 subjects from 22 centers between March 2006 and December 2010 in the UK Biobank were analyzed (Figure 1). Confirmed COVID-19 infection was defined as at least 1 positive test result, 60,446 subjects of whom received COVID-19 test results up to February 24, 2021, with 14,877 subjects confirmed positive for COVID-19 infection (Figure 2). Subjects who did not undergo COVID-19 testing were deemed to have a negative COVID-19 test result. Subjects who received positive COVID-19 test results were classified into two categories: death due to and not due to COVID-19. These data were used to study the risk of COVID-19 associated mortality. Ethical approval was obtained from the North West Multi-Centre Research Ethics Committee (REC reference: 16/NW/0274).

### Categories of Mental Disorders

Mental disorders were classified according to International Classification of Diseases, Version 10 (ICD-10) terms from UK Biobank data field 41,270, these included dementia (ICD-10 codes F00-F04, and G30), psychotic disorder (codes F20-F29), substance use disorder (codes F10-F19), bipolar disorder (codes F30-F31), anxiety (codes F40-F41), and depression (codes F32-F33). Dementia included Alzheimer disease, vascular dementia, mixed dementia, and secondary dementia. Depression and anxiety were grouped according to diagnosis in middle-age (<60 years) and late-life (≥60 years).

### Categories of Confounding Factors

Confounding factors included demographic variables (e.g., age, sex, education level, and ethnicity) and comorbidities (e.g., obesity, cerebrovascular disease, diabetes, hypertension, coronary artery disease, cancers, and respiratory diseases).

Education level (Field ID 6138) was classified into two categories depending on the presence of college or university degree. The definition of obesity was a body mass index (BMI) >30 kg/m^2^. The definition of diabetes included self-reported type 1 or type 2 diabetes and a primary or secondary hospital diagnosis related to diabetes (ICD-10 codes E10-E14.9). Respiratory disease was defined as pulmonary embolism (code I26), other pulmonary heart diseases (code I27), influenza (codes J09-J11), pneumonia (codes J12-J18), acute bronchitis (code J20), acute bronchiolitis (code J21), unspecified acute lower respiratory infection (code J22), other diseases of the upper respiratory tract (codes J30-J39), chronic lower respiratory diseases (codes J40-J47), lung diseases due to external agents (codes J60-J70), other respiratory diseases principally affecting the interstitium (codes J85-86), and other diseases of the pleura and respiratory system (ICD-10 codes J90-99).

### Statistical Analysis

Continuous variables are presented as mean ± SD, and categorical variables are presented as a number (percentage). We used unpaired *t*-test and χ^2^-test to compare differences between groups where appropriate.

Logistic regression analysis was used to evaluate associations between mental disorders and risk of COVID-19 infection and associated mortality (among subjects who received positive COVID-19 test results). A univariate model (Model 1) and multivariate models (Model 2–4, adjusted for several confounding factors) were applied to determine the Odd Ratios (ORs) and 95% confidence intervals (95% CIs). Two-tailed *p* < 0.05 were considered statistically significant.

## Results

### Sample Characteristics

The demographic characteristics of the study population are presented in Table 1. Total of 8.9% subjects (*n* = 42,264) had a diagnosis of mental disorders, among whom 19.8% subjects (*n* = 8,378) had a chance to receive the COVID-19 test (21.9% subjects had a positive COVID-19 test result; *n* = 1,838). Among the 1,838 subjects, 8.9% (*n* = 164) deaths due to the COVID-19. However, only 12.1% subjects (*n* = 52,064) had a chance to receive the COVID-19 test among the remaining subjects who did not have a diagnosis of mental disorders (*n* = 4,31,694).

**Table 1:** Characteristics of UK Biobank cohort.

**Characteristics**	**COVID-19 (*n* = 14,874)**	**Non-COVID-19 (*n* = 4,59,084)**	***p-*value**	**Death due to COVID-19 (*n* = 664)**	**Non-causing death due to COVID-19 (*n* = 14,210)**	***p-*value**
Age to December 2020 (years), mean ± SD	65.5 ± 8.6	68.2 ± 8.1	<0.001	74.9 ± 5.7	65.0 ± 8.5	<0.001
Sex (male), *N* (%)	7,031 (47.3)	2,05,036 (44.7)	<0.001	442 (66.6)	6589 (46.4)	<0.001
Education (no degree), *N* (%)	10,864 (74.8)	2,99,158 (66.5)	<0.001	529 (82.9)	10335 (74.4)	<0.001
Ethnicity (white), *N* (%)	13,263 (89.7)	4,32,065 (94.6)	<0.001	605 (91.8)	12658 (89.6)	0.064
Obesity (BMI ≥30 kg/m^2^), *N* (%)	4,563 (31.0)	1,09,020 (23.9)	<0.001	283 (43.6)	4280 (30.4)	<0.001
Cerebrovascular disease, *N* (%)	442 (3.0)	8,820 (1.9)	<0.001	69 (10.4)	373 (2.6)	<0.001
Diabetes, *N* (%)	1,296 (8.7)	24,958 (5.4)	<0.001	171 (25.8)	1125 (7.9)	<0.001
Hypertension, *N* (%)	3,660 (24.6)	96,523 (21.0)	<0.001	363 (54.7)	3297 (23.2)	<0.001
Coronary artery disease, *N* (%)	1,241 (8.3)	31,102 (6.8)	<0.001	153 (23.0)	1088 (7.7)	<0.001
Cancer*, N* (%)	1,688 (11.3)	59,069 (12.9)	<0.001	139 (20.9)	1549 (10.9)	<0.001
Respiratory disease, *N* (%)	3,078 (20.7)	72,450 (15.8)	<0.001	249 (37.5)	2829 (19.9)	<0.001
Psychotic disorders, *N* (%)	51 (0.3)	1,172 (0.3)	0.038	6 (0.9)	45 (0.3)	0.011
Substance use disorder, *N* (%)	803 (5.4)	18,733 (4.1)	<0.001	61 (9.2)	742 (5.2)	<0.001
Bipolar affective disorder, *N* (%)	64 (0.4)	1,267 (0.3)	<0.001	11 (1.7)	53 (0.4)	<0.001
Dementia, *N* (%)	291 (2.0)	1,715 (0.4)	<0.001	62 (9.3)	229 (1.6)	<0.001
Alzheimer disease	226 (1.5)	1,251 (0.3)	<0.001	47 (7.1)	179 (1.3)	<0.001
Vascular dementia	34 (0.2)	171 (0.0)	<0.001	5 (0.8)	29 (0.2)	0.004
Mixed dementia	7 (0.0)	33 (0.0)	<0.001	0 (0.0)	7 (0.0)	<0.001
Secondary dementia	72 (0.5)	503 (0.1)	<0.001	18 (2.7)	54 (0.4)	0.57
Anxiety, *N* (%)	470 (3.2)	9,577 (2.1)	<0.001	34 (5.1)	436 (3.1)	0.003
Middle-aged anxiety	118 (0.8)	1,685 (0.4)	<0.001	0 (0.0)	118 (0.8)	0.018
Late-life anxiety	352 (2.4)	7,892 (1.7)	<0.001	34 (5.1)	318 (2.2)	<0.001
Depression, *N* (%)	782 (5.3)	15,710 (3.4)	<0.001	65 (9.8)	717 (5.0)	<0.001
Middle-age	186 (1.3)	3,215 (0.7)	<0.001	4 (0.6)	182 (1.3)	0.12
Late-life	596 (4.0)	12,495 (2.7)	<0.001	61 (9.2)	535 (3.8)	<0.001
Depression comorbid with anxiety, *N* (%)	204 (1.4)	3,346 (0.7)	<0.001	16 (2.4)	188 (1.3)	0.019
Middle-age	45 (0.3)	715 (0.2)	<0.001	0 (0.0)	45 (0.3)	0.15
Late-life	159 (1.1)	2,631 (0.6)	<0.001	16 (2.4)	143 (1.0)	0.001

In general, subjects diagnosed with a mental disorder had higher COVID-19 infection and COVID-19-associated mortality rates than those without a mental disorder; this was not true of COVID-19 morbidity rates in subjects with secondary dementia, middle-aged depression, and middle-aged depression comorbid middle-aged anxiety.

Of the 14,874 subjects in the database who were positive for COVID-19, 664 (4.5%) subjects died due to the COVID-19. Among subjects diagnosed with a mental disorder, the highest rates of COVID-19 infection were among subjects with substance use disorder (5.4%), depression (5.3%), anxiety (3.2%), and dementia (2.0%), whereas subjects with psychotic disorder (0.3%), and bipolar affective disorder (0.4%) had the lowest rates. Among cases of COVID-19-related death, depression (9.8%), dementia (9.3%), substance use disorder (9.2%), and anxiety (5.1%) were the most common mental disorders; there were no deaths from COVID-19 among subjects with mixed dementia, middle-aged anxiety and middle-aged depression comorbid with anxiety.

### Association Between Mental Disorders and COVID-19 Incidence

Subjects with a diagnosis of dementia (OR [95% CI]: 6.43 [5.62, 7.35], *p* < 0.001), anxiety (1.29 [1.17, 1.42], *p* < 0.001), or depression (1.22 [1.13, 1.31], *p* < 0.001) had a significantly higher probability of COVID-19 infection than those without a mental disorder after adjusting for age, sex, education level, ethnicity, BMI, cerebrovascular disease, diabetes, hypertension, coronary artery disease, cancer, and respiratory disease, with the strongest effect observed for Alzheimer disease (7.39 [6.36, 8.60], *p* < 0.001) among dementia subtypes (Table 2). Furthermore, we also analyzed the COVID-19 risk of these mental disorders among the subjects who received COVID-19 test. Subjects with a diagnosis of dementia (2.89 [2.48, 3.36], *p* < 0.001) still had a significantly higher probability of COVID-19 infection than those without a mental disorder, and all dementia subtypes showed more than 2-fold risk of COVID-19 infection (Alzheimer disease, 2.94 [2.47, 3.49], *p* < 0.001; vascular dementia, 3.88 [2.45, 6.14], *p* < 0.001; mixed dementia, 4.58 [1.68, 12.50], *p* = 0.011; secondary dementia, 2.40 [1.78, 3.24], *p* < 0.001).

**Table 2:** Logistic regression analysis of the association between mental disorders and risk of COVID-19.

**Variables**	**Risk of infection for COVID-19**							
	**Model 1**		**Model 2**		**Model 3**		**Model 4**	
	**OR (95%CI)**	***p*-value**	**OR (95%CI)**	***p*-value**	**OR (95%CI)**	***p*-value**	**OR (95%CI)**	***p*-value**
Psychotic disorders	1.34 (1.02–1.78)	0.039	1.20 (0.91–1.59)	0.2	0.93 (0.68–1.27)	0.66	0.82 (0.60–1.12)	0.21
Substance use disorder	1.34 (1.25–1.44)	<0.001	1.29 (1.20–1.39)	<0.001	1.20 (1.12–1.30)	<0.001	1.03 (0.95–1.11)	0.52
Bipolar affective disorder	1.56 (1.21–2.01)	0.001	1.50 (1.17–1.94)	0.002	1.41 (1.08–1.83)	0.01	1.23 (0.94–1.59)	0.13
Dementia	5.32 (4.70–6.03)	<0.001	7.82 (6.89–8.88)	<0.001	7.54 (6.61–8.60)	<0.001	6.43 (5.62–7.35)	<0.001
Alzheimer disease	5.65 (4.90–6.51)	<0.001	8.50 (7.36–9.83)	<0.001	8.29 (7.14–9.63)	<0.001	7.39 (6.36–8.60)	<0.001
Vascular dementia	6.15 (4.25–8.89)	<0.001	8.80 (6.07–12.74)	<0.001	8.56 (5.85–12.52)	<0.001	5.81 (3.94–8.55)	<0.001
Mixed dementia	6.55 (2.90–14.81)	<0.001	9.60 (4.23–21.75)	<0.001	9.63 (4.19–22.12)	<0.001	6.33 (2.70–14.82)	<0.001
Secondary dementia	4.44 (3.46–5.68)	<0.001	6.08 (4.74–7.80)	<0.001	5.78 (4.46–7.48)	<0.001	4.42 (3.40–5.75)	<0.001
Anxiety	1.53 (1.39–1.68)	<0.001	1.59 (1.44–1.74)	<0.001	1.51 (1.37–1.66)	<0.001	1.29 (1.17–1.42)	<0.001
Middle-age	2.17 (1.80–2.62)	<0.001	1.39 (1.15–1.68)	0.001	1.28 (1.06–1.55)	0.012	1.10 (0.90–1.33)	0.35
Late-life	1.39 (1.24–1.54)	<0.001	1.66 (1.49–1.85)	<0.001	1.60 (1.43–1.78)	<0.001	1.36 (1.22–1.52)	<0.001
Depression	1.57 (1.46–1.69)	<0.001	1.54 (1.43–1.66)	<0.001	1.43 (1.32–1.54)	<0.001	1.22 (1.13–1.31)	<0.001
Middle-age	1.80 (1.55–2.08)	<0.001	1.15 (0.99–1.33)	0.073	1.01 (0.87–1.18)	0.89	0.86 (0.74–1.01)	0.06
Late-life	1.49 (1.37–1.62)	<0.001	1.71 (1.58–1.86)	<0.001	1.62 (1.48–1.76)	<0.001	1.38 (1.26–1.50)	<0.001
Depression comorbid with anxiety	1.89 (1.64–2.18)	<0.001	1.86 (1.61–2.14)	<0.001	1.73 (1.50–2.00)	<0.001	1.42 (1.23–1.65)	<0.001
Middle-age	1.95 (1.44–2.63)	<0.001	1.25 (0.92–1.69)	0.15	1.10 (0.80–1.50)	0.56	0.90 (0.66–1.23)	0.50
Late-life	1.88 (1.60–2.20)	<0.001	2.14 (1.82–2.52)	<0.001	2.04 (1.73–2.40)	<0.001	1.67 (1.42–1.97)	<0.001

*Model 1 unadjusted.*

*Model 2 analyses were adjusted for age and sex.*

*Model 3 analyses were further adjusted for education, ethnicity, and BMI.*

*Model 4 Analyses were further adjusted for cerebrovascular disease, diabetes, hypertension, coronary artery disease, cancer, and respiratory disease*.

Subjects with a diagnosis of psychotic disorder did not have a higher risk of COVID-19 infection (*p* = 0.21) or COVID-19-related death (*p* = 0.23) than those without a mental disorder (Tables 2, 3). Substance use disorder and bipolar affective disorder were associated with a significantly higher risk of COVID-19 infection compared to absence of a mental disorder after adjusting for age, sex, education level, ethnicity, and BMI in model 3; however, after further adjusting for medical comorbidities (model 4), the higher risk was not significant. In the finally model, subjects with middle-age anxiety, or middle-age depression did not have a higher risk of COVID-19 infection; those with anxiety did not have a higher risk of death from COVID-19; and those with substance use disorder did not have higher risks for COVID-19 infection and associated mortality than subjects without these mental disorders.

**Table 3:** Logistic regression analysis of the association between mental disorders and risk of death due to COVID-19.

**Variables**	**Death risk due to COVID-19**							
	**Model 1**		**Model 2**		**Model 3**		**Model 4**	
	**OR (95% CI)**	***p*-value**	**OR (95% CI)**	***p*-value**	**OR (95%CI)**	***p*-value**	**OR (95% CI)**	***p*-value**
Psychotic disorders	2.87 (1.22–6.75)	0.016	2.77 (1.10–7.01)	0.031	2.29 (0.82–6.37)	0.11	1.88 (0.68–5.23)	0.23
Substance use disorder	1.84 (1.40–2.41)	<0.001	1.60 (1.20–2.14)	0.001	1.59 (1.18–2.16)	0.003	1.23 (0.90–1.69)	0.20
Bipolar affective disorder	4.50 (2.34–8.66)	<0.001	4.50 (2.18–9.27)	<0.001	4.77 (2.31–9.87)	<0.001	3.69 (1.78–7.67)	<0.001
Dementia	6.29 (4.70–8.42)	<0.001	2.03 (1.49–2.76)	<0.001	2.25 (1.63–3.10)	<0.001	2.06 (1.49–2.85)	<0.001
Alzheimer disease	5.97 (4.29–8.32)	<0.001	1.85 (1.31–2.63)	0.001	2.18 (1.52–3.11)	<0.001	2.13 (1.48–3.06)	<0.001
Vascular dementia	3.71 (1.43–9.62)	0.007	1.20 (0.45–3.18)	0.71	1.33 (0.50–3.52)	0.57	0.91 (0.33–2.45)	0.84
Secondary dementia	7.30 (4.26–12.53)	<0.001	2.69 (1.53–4.75)	0.001	2.68 (1.47–4.88)	0.001	2.09 (1.13–3.85)	0.019
Anxiety	1.71 (1.19–2.44)	0.003	1.57 (1.07–2.29)	0.02	1.56 (1.06–2.31)	0.024	1.27 (0.85–1.89)	0.24
Late-life anxiety	2.36 (1.64–3.39)	<0.001	1.61 (1.10–2.35)	0.014	1.60 (1.09–2.37)	0.018	1.30 (0.87–1.94)	0.20
Depression	2.04 (1.56–2.67)	<0.001	2.08 (1.57–2.77)	<0.001	2.03 (1.51–2.72)	<0.001	1.57 (1.16–2.13)	0.004
Late-life	2.59 (1.96–3.41)	<0.001	1.97 (1.47–2.63)	<0.001	1.90 (1.40–2.57)	<0.001	1.46 (1.07–2.00)	0.017
Depression comorbid with anxiety	1.84 (1.10–3.09)	0.02	1.87 (1.09–3.24)	0.024	1.67 (0.93–2.97)	0.08	1.31 (0.73–2.36)	0.36
Late-life	2.43 (1.44–4.10)	0.001	1.92 (1.11–3.32)	0.02	1.71 (0.95–3.05)	0.07	1.35 (0.75–2.42)	0.32

*Model 1 unadjusted.*

*Model 2 analyses were adjusted for age and sex.*

*Model 3 analyses were further adjusted for education, ethnicity, and BMI.*

*Model 4 Analyses were further adjusted for cerebrovascular disease, diabetes, hypertension, coronary artery disease, cancer, and respiratory disease*.

Subjects with a diagnosis of a dementia (2.06 [1.49, 2.85], *p* < 0.001) or a depression (1.57 [1.16, 2.13], *p* = 0.004) had a significantly higher risk of death from COVID-19 than those without a mental disorder. A diagnosis of depression comorbid with anxiety increased probability of COVID-19 infection (depression comorbid with anxiety, 1.42 [1.23, 1.65], *p* < 0.001; depression, 1.22 [1.13, 1.31], *p* < 0.001; anxiety, 1.29 [1.17, 1.42], *p* < 0.001) but not COVID-19–related death of depression comorbid with anxiety (1.31 [0.73, 2.36], *p* = 0.36) compared to depression (1.57 [1.16, 2.13], *p* = 0.004) or anxiety (1.27 [0.85, 1.89], *p* = 0.24) alone.

## Discussion

This study of a large sized UK Biobank cohort yielded three novel findings. First, we document that subject diagnosed with a mental disorder had a significantly higher risk of COVID-19 infection and worse outcome (COVID-19-related death) than those without a mental disorder. Second, among dementia subtypes, Alzheimer disease exhibited the highest risk of COVID-19 infection (7.39-fold risk) and COVID-19–related death (2.13-fold risk). Mixed dementia and vascular dementia did not increase the risk of COVID-19-related death. Third, late-life anxiety increased the risk of COVID-19 infection, and subjects with late-life depression had a higher risk of COVID-19 infection and a worse outcome from COVID-19 than those without late-life depression. Moreover, late-life depression comorbid with anxiety significantly increased the risk of COVID-19 infection compared to either disorder alone. However, middle-aged anxiety, middle-aged depression, and depression comorbid with anxiety were not associated with elevated risks of COVID-19 infection and COVID-19-related death.

The most striking and strongest risk factors for COVID-19 infection and associated death was dementia. In particular, subjects with Alzheimer disease had the highest risks of COVID-19 infection and associated mortality among all dementia subtypes. This finding was confirmed by a previous study (17). There are a few possible explanations for this finding. Firstly, SARS-CoV-2 has been detected in the central nervous system of patients with dementia (18) and may cause brain damage and dysfunction (19). Patients with severe neurological dysfunction and injury are more likely to develop pulmonary diseases that further worsen clinical outcomes, in what is known as the brain-lung-brain axis theory (20). Secondly, the risk genotype for dementia was shown to predict the severity of COVID-19 (21) and risk of COVID-19–related death (22). Thirdly, patients with dementia often require the support of others to communicate their needs; however, the COVID-19 pandemic has reduced the availability of social support services for these patients (23).

Our results also suggest that anxiety and depression were only risk factors in subjects diagnosed with these disorders in late-life but not in middle age. The negative effects of anxiety and depression on immunity are not fully understood. Age is a known risk factor for COVID-19 infection (24). The immune system undergoes numerous age-related changes in a process known as immune senescence (25); we speculated that this is the reason for the higher risks of COVID-19 infection and associated mortality in late-life anxiety and late-life depression relative to middle-aged anxiety and middle-aged depression. Late-life depression has been linked to an elevated risk of respiratory conditions (26) and is a risk factor for or early symptom of dementia (27), but not at younger ages (28). These findings may also explain why anxiety and depression were found to be risk factors for COVID-19 infection and associated mortality only in subjects diagnosed at older ages.

The strengths of this study are concerning the COVID-19 risk and associated mortality for dementia subtypes and age stratification for depression and anxiety with a large sized cohort. However, there are several limitations that should be addressed. First, subjects in the UK Biobank have a restricted age range, and therefore our data could not represent the whole population. Second, individuals identified with positive COVID-19 tests from the UK Biobank cohort may represent the hospitalized and severe cases, which might limit the generalization. Third, subjects who did not undergo COVID-19 testing were deemed to have a negative COVID-19 test result, and we did not know if some subjects were tested but not captured in our dataset. Fourth, some medical co-morbidities may not be listed in the hospital diagnosis, and therefore the number of reported medical comorbidities may be lower than the actual number.

In conclusion, our findings indicate that public health measures for COVID-19 such as vaccinations should prioritize individuals with mental disorders, especially those with both COVID-19 infection and dementia or depression. Future studies should explore the risk factors for COVID-19 in individuals with late-life-related diseases (e.g., dementia), given the increasing prevalence of these disorders in the global population.

## Data Availability Statement

Publicly available datasets were analyzed in this study. This data can be found here: UKBIOBANK.

## Ethics Statement

The studies involving human participants were reviewed and approved by ethical approval was obtained from the North West Multi-Centre Research Ethics Committee (REC reference: 16/NW/0274). The patients/participants provided their written informed consent to participate in this study.

## Author Contributions

X-jD and YW had the idea for and designed this study, had full access to all the data in this study, take responsibility for the integrity of the data and the accuracy of the data analysis, critically revised the manuscript for important intellectual content and gave final approval for the version to be published, drafted the paper, and did the analysis. YW, YY, LR, YS, and WT takes responsibility for double check of the data analysis. All authors agree to be accountable for all aspects of the work in ensuring that questions related to the accuracy or integrity of any part of the work are appropriately resolved.

## Conflict of Interest

The authors declare that the research was conducted in the absence of any commercial or financial relationships that could be construed as a potential conflict of interest.

## Publisher's Note

All claims expressed in this article are solely those of the authors and do not necessarily represent those of their affiliated organizations, or those of the publisher, the editors and the reviewers. Any product that may be evaluated in this article, or claim that may be made by its manufacturer, is not guaranteed or endorsed by the publisher.
